# An Unusual Complication Following Typhoid Fever

**Published:** 1906-06

**Authors:** J. E. Sommerfield

**Affiliations:** Atlanta, Ga.


### AN UNUSUAL COMPLICATION FOLLOWING TYPHOID FEVER; REPORT OF CASE.

BY J. E. SOMMERFIELD, M.D.,

ATLANTA, GA.

Abscess of the liver is seen only occasionally in this section of the country, but when occurring as a complication of typhoid fever, it is exceedingly rare, and for that reason, I am sure that the report of the following case will be of interest to the members of this Association.

A. S., male, white, age thirty-nine.

*Family History*—Both parents living and in good health. Two of his brothers have had severe attacks of typhoid fever in recent years.

*Personal History.*—He has always enjoyed the best of health until eight years ago, when he had a chronic diarrhoea of such severity that he was unable to attend to his business for nearly three years. During the past five years, however, his health has been good, and he has experienced no bowel trouble.

He was first seen on August 20, 1905, when he stated that he felt badly for at least two weeks, although he did not take to his bed. On examination, the following symptoms were noted:

Temperature 102.8 F., pulse 94, headache, tongue dry and furred, a slight cough, abdomen somewhat tympanitic, tenderness in the right iliac region, five or six rose-colored spots on the abdomen, constipation. Diagnosis: Typhoid fever.

To confirm the diagnosis, Russo's test was used. This test has been suggested as a substitute for Ehrlich's diazo reaction. It is much simpler and seems to be equally reliable. As it may probably be unfamiliar to some of you, a description of the test may not be out of place here.

In typhoid fever it affords more information than the diazo test, as it indicates the exact phase of the disease. The reagent used is a 1-1000 aqueous solution of Methylene Blue. To a drachm of urine in a test-tube, add four drops of the Methylene Blue solution, and if the reaction be positive, the fluid turns to a mint or emerald-green color. A light green or bluish green represents a negative reaction. This reaction can be seen as early as the second or third day of the disease. As the fever reaches its height the dark-green color becomes more marked, gradually turning to a light-green, and finally a blue, as convalescence is established or the emerald-color persists until death. The reaction was positive in eleven out of twelve cases seen by me.

To return to the patient. On the twentieth day of treatment, the temperature returned to the normal and remained so for six days, when a relapse occurred which lasted two weeks. Just about this time he complained of a dull pain in the region of the liver, but a careful examination showed no enlargement. In the course of four or five days the pain became quite intense, the temperature was normal each morning, but at night rose to 103, followed by a profuse perspiration. Gradually an enlargement of the liver was noticed with a marked bulging of the intercostal spaces. Abscess of the liver was diagnosed, and Dr. H. F. Harris called in consultation, and the diagnosis was confirmed by him. On aspiration pus was found. It was examined microscopically by Dr. Harris, but with a negative result. Immediate operation was advised, and on the following day an incision was made in the ninth intercostal space and a large quantity of pus was evacuated. No rib resected. The cavity was not irrigated, but large drainage tubes inserted. For several days the patient was in a critical condition, then rallied, and in a short time made a complete recovery. At the present time he is in the best of health, and weighs more than ever before.

*803-4 Century Building.*

